# Prevalence, Risk Factors, and Postoperative Infection Rates of Blood Transfusion in Lumbar Spinal Fusion Surgery: A Nationwide Population-Based Study

**DOI:** 10.3390/jcm13164867

**Published:** 2024-08-18

**Authors:** Si Young Park, Taewook Kang, Woong Kyo Jeong, Ji Eun Song

**Affiliations:** 1 Department of Orthopedics, Yonsei University College of Medicine, Seoul 03722, Republic of Korea; drspine90@gmail.com; 2 Department of Spine Surgery, Cheil Orthopedic Hospital, Seoul 06075, Republic of Korea; 3 Department of Orthopedics, Anam Hospital, Korea University College of Medicine, Seoul 02841, Republic of Korea; drshoulder@korea.ac.kr; 4 Department of Biostatistics, Korea University College of Medicine, Seoul 02841, Republic of Korea; sjieun7884@korea.ac.kr

**Keywords:** lumbar spinal fusion, blood transfusion, infection

## Abstract

**Study Design:** Retrospective cohort study. **Objectives:** Effects of blood loss that requires blood transfusion after lumbar spinal fusion remain an important issue. Blood transfusions are used commonly in cases of significant blood loss in lumbar spinal fusion but are associated with adverse effects. The objective was to assess the rate of blood transfusion and the associated risk after lumbar spinal fusion from 2013 to 2018. **Methods:** In this nationwide population-based cohort study, the Korean Health Insurance Review and Assessment Service database was reviewed retrospectively from 2013 to 2018. Data were extracted from patients who underwent lumbar spinal fusion without history of lumbar spinal surgery in the preceding year. The primary outcome was the rate of blood transfusion within 1 week of surgery. In addition, the risk factors for blood transfusion and the rate of postoperative infection were evaluated. **Results:** A total of 188,581 patients underwent lumbar spinal fusion between 2013 and 2018. A significant decline in blood transfusions was observed during the study period (56.38–47.51%). The presence of comorbidities was associated with an increased risk of blood transfusion. Patients who underwent the posterior approach were more likely to receive blood transfusion than patients who underwent the anterior or anterior and posterior approach. Receiving blood transfusion was associated with postoperative infection. **Conclusions:** In the present study, the prevalence, risk factors, and postoperative infection rates associated with blood transfusion in lumbar spinal fusion were identified. Spine surgeons should consider these risk factors in patients at high risk of blood transfusion.

## 1. Introduction

Lumbar spinal fusion is performed widely for the treatment of various conditions, such as stenosis, spondylolisthesis, deformity, tumor, trauma, and infection [1,2]. However, lumbar spinal fusion can be associated with significant intraoperative blood loss, and some patients require perioperative blood transfusions [3]. Recent studies have reported that the rate of blood transfusion has been increasing in patients undergoing lumbar spinal fusion, doubling in the past decade with a rate increasing from 4% to 8% in the United States from 2000 to 2009 [4,5].

Intraoperative blood loss requires blood transfusions to avoid perioperative anemia, which is a risk factor for perioperative morbidity and mortality [6]. However, a balance must be achieved between the risk of anemia and the benefits from blood transfusion while considering the effect on perioperative outcomes [7,8]. Patients who become hemodynamically unstable due to intraoperative blood loss are at increased risk of serious ischemic conditions, such as myocardial infarction or cerebrovascular disease. Blood transfusions are associated with an increased risk of postoperative complications, including blood-borne infections, immunosuppression, allergic or febrile reactions, acute lung injury, and wound complications [9,10]. Several authors have found a correlation between blood transfusion and postoperative infection [11,12]. These complications of blood transfusion result in significantly increased patient morbidity, mortality, and length of hospital stay, and poor postoperative functional recovery [13,14,15]. It is also an important issue that blood transfusions can place a significant economic burden on patients, their families, and the healthcare system [16,17]. In addition, because blood is a limited source, continuous efforts to prevent unnecessary blood transfusions should be made [18,19,20,21].

Recently, several authors have reported significant changes in blood transfusion trends over the past two decades, such as decreases in transfusion thresholds, hemoglobin at discharge, and the number of blood transfusions during surgery [22]. There are ongoing efforts to conserve blood and decrease blood transfusions for patient safety and to reduce costs [5]. Determining the risk factors for blood transfusion could better identify patients at risk of perioperative blood transfusion and allow for better optimization and management to reduce the need and number of blood transfusions and the associated complications [23]. The detection of patients with risk factors could be optimized prior to surgery. Nonmodifiable risk factors could be included in the informed consent form, and whether to proceed with surgery can be discussed with patients prior to lumbar spinal fusion.

In the present population-based study, the prevalence of blood transfusion and the risk factors for predicting blood transfusion were assessed in a large number of patients undergoing lumbar spinal fusion using a nationwide administrative database. The primary objective was to determine the prevalence of blood transfusion patients who underwent lumbar spinal fusion in Korea from 2013 to 2018. The second objective was to identify the risk factors for blood transfusion in patients undergoing lumbar spinal fusion.

## 2. Methods

### 2.1. Database

Data from the Korean Health Insurance Review and Assessment Service (HIRA) database were collected. All Korean citizens are required to register for the Korean National Health Insurance Service (KNHIS), to which all hospitals submit their data on patient diagnosis and treatment costs. All medical treatments are tracked through the HIRA system without exception. The KNHIS enables both population-based studies and longitudinal analyses.

This nationwide, population-based, retrospective cohort study was approved by HIRA and the Institutional Review Board of Korea University Anam Hospital (IRB no. 2019AN0340). All identifiable personal information was removed and anonymized data for research purposes were used. Therefore, the requirement for informed consent was waived by the IRB of our institution.

The HIRA database includes patient demographics; clinical information such as disease diagnoses, drug prescriptions, and procedures; as well as hospital information. The diagnoses were recorded according to the International Classification of Diseases, 10th Revision (ICD-10) codes. The operations and procedures were identified based on the Korean Electronic Data Interchange (EDI) codes.

Primary lumbar spinal fusions performed from January 2013 to December 2018 in Korea were identified using EDI codes of anterior fusion [lumbar spine] (N0466), posterior fusion [lumbar spine] (N0469), posterior fusion using cage [lumbar spine] (N2470), anterior fusion [lumbar spine]—complex (N1466), posterior fusion [lumbar spine]—complex (N1469), and posterior fusion using cage [lumbar spine]—complex (N1460).

### 2.2. Study Sample, Definitions, and Outcomes

Patients older than 20 years who underwent lumbar spinal fusion between 1 January 2013 and 31 December 2018 were selected from the HIRA database. By including only new interventions without prior lumbar spinal surgery during the preceding 1 year, the influence of previous lumbar spinal surgeries was eliminated.

Blood transfusion was defined based on procedure codes for transfusion within 1 week of the primary procedure (EDI: X1001, X1002, X2011, X2012, X2021, X2022, X2031, X2032, X2041, X2051, X2052, X2061, X2062, X2071, X2072, X2081, X2082, X2091, X2092, X2101, X2102, X2111, X2112, X2121, X2122, X2131, X2132, X2141, X2142, X2501, X2502, X2504, X2511, X2512, X2513, X2514, X2515, and X2516).

Infection was defined as either an additional procedure code for debridement or a diagnostic code for infection recorded within 1 month of surgery (EDI: SC021, SC022, SC023, SC024, SC025, SC026, SC027, N0841, N0842, N0843, N0844, N2471, N2472, ICD-10: M46.2, M46.3, M46.4, M46.5, M49.0, M49.1, M49.2, M49.3, T81.3, T81.4, T81.9, T84.6, or T84.7).

### 2.3. Confounding Factors (Potential Risk Factors)

Age, sex, presence of comorbidities, insurance type (National Health Insurance vs. Medical Aid), hospital size (tertiary referral hospital vs. general hospital vs. hospital vs. private clinic), hospital location (metropolitan vs. urban vs. rural), surgical approach (anterior vs. posterior vs. anterior and posterior), and presence of osteotomy (EDI: N0303) were evaluated as confounding factors.

The ICD-10 codes were used to evaluate the presence of comorbidities. The comorbidities in each patient were identified by screening the presence of primary or secondary diagnostic codes from any hospital visit within 1 year before or after the date of surgery. Comorbidities were assessed using the Charlson Comorbidity Index, a well-established method for identifying comorbidities in administrative databases.

### 2.4. Statistical Analysis

Data on baseline characteristics were presented as frequencies and percentages. Baseline characteristics of the simple and complex groups were compared using χ2 tests. To identify the significant risk factors for blood transfusion and postoperative infection, univariate and multivariate logistic regressions were conducted. The results are presented as odds ratios (ORs) with 95% confidence intervals (CIs). The Mann–Kendall method was used to analyze the time trend of blood transfusion prevalence during the study period. A *p*-value < 0.05 was considered statistically significant. Statistical analyses were performed using SAS software version 9.4 (SAS Institute, Cary, NC, USA) and R Statistical software version 3.3.3 (Foundation for Statistical Computing, Vienna, Austria).

## 3. Results

### 3.1. Overall Outcome

**Figure 1:**
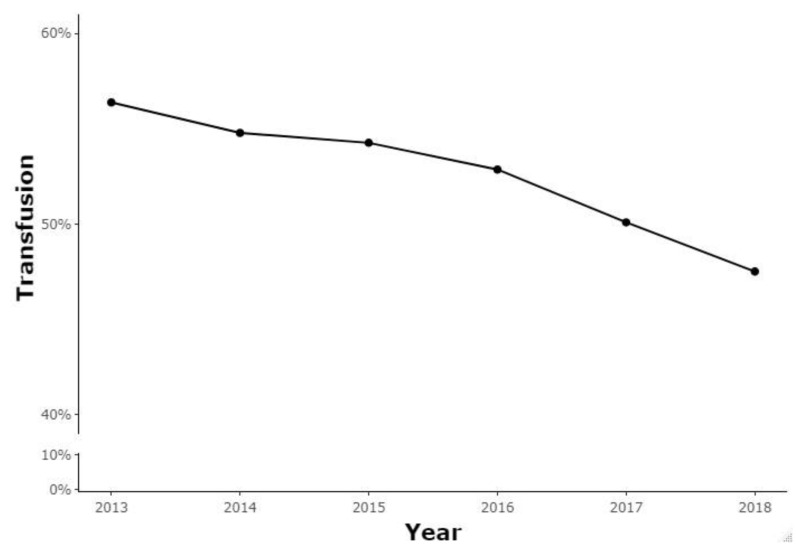
Blood transfusion rates among lumbar spinal fusion patients.

A total of 190,200 patients underwent lumbar spinal fusion in Korea from 1 January 2013 to 31 December 2018. Patients < 20 years of age, patients with previous lumbar spinal surgery in the preceding 1 year, and patients with fracture, neoplasm, or infection were excluded. In the remaining 188,581 patients, the overall blood transfusion rate was 52.63% (99,247/188,581). From 2013 to 2018, a steady decrease in the annual prevalence of blood transfusion was observed (56.38–47.51%, *p* = 0.085; Figure 1, Table 1). Table 2 shows detailed information on the baseline characteristics, hospital characteristics, and comorbidities of the transfusion and no-transfusion groups of patients who underwent lumbar spinal fusion.

### 3.2. Risk Factors for Blood Transfusion

Multivariate logistic regression analysis was performed to identify independent risk factors for blood transfusion. Table 3 shows the ORs and 95% CIs for blood transfusion-related risk factors in lumbar spinal fusion.

The risk of blood transfusion was higher in patients with advanced age (OR, 1.082, 95% CI, 1.022–1.145 for 50–59 years of age; OR, 1.541, 95% CI, 1.457–1.63 for 60–69 years of age; OR, 2.391, 95% CI, 2.258–2.531 for ≥70 years of age), female patients (OR, 1.772; 95% CI, 1.737–1.807), and patients using Medical Aid (OR, 1.543; 95% CI, 1.478–1.592); for small-sized hospitals (OR, 1.115; 95% CI, 1.086–1.146 for general hospitals; OR, 1.03; 95% CI, 1.005–1.055 for hospitals; OR, 4.148; 95% CI, 3.583–4.801 for private clinics), hospitals in rural areas (OR, 1.211; 95% CI, 1.186–1.236 for urban area; OR, 3.506; 95% CI, 2.682–4.582 for rural area), and patients who underwent the posterior approach (anterior: OR, 0.511; 95% CI, 0.48–0.545; anterior and posterior: OR, 0.426; 95% CI, 0.412–0.44); and with the performance of osteotomy (OR, 37.019; 95% CI, 21.227–66.558), the presence of myocardial infarction (OR, 1.149; 95% CI, 1.076–1.226), the presence of diabetes (OR, 1.032; 95% CI, 1.01–1.056), the presence of kidney disease (OR, 1.323; 95% CI, 1.257–1.393), the presence of renal disease (OR, 1.323; 95% CI, 1.257–1.393), the presence of severe liver disease (OR, 1.387; 95% CI, 1.244–1.546), and the presence of metastatic solid tumor (OR, 2.166; 95% CI, 1.937–2.421).

### 3.3. Prevalence of Postoperative Infection

The overall infection rate was 9.99% (18,839/188,581) in patients who underwent lumbar spinal fusion. The rate of infection was 12.47% (12,374/99,247) in the transfusion group and 7.24% (6465/89,334) in the no-transfusion group. Patients who received blood transfusion were more likely to experience postoperative infection compared with those who did not receive blood transfusion (OR, 1.876; 95% CI, 1.815–1.939).

Table 4 shows the ORs and 95% CIs for the variables other than blood transfusion associated with postoperative infection in lumbar spinal fusion. The risk of infection was lower in patients who were female (OR, 0.72; 95% CI, 0.698–0.743), and lower in patients who underwent osteotomy (OR, 1.256; 95% CI, 0.969–1.629), had diabetes (OR, 1.075; 95% CI, 1.037–1.114), or had diabetes complications (OR, 1.077; 95% CI, 1.031–1.124).

## 4. Discussion

In the present study, a large database was analyzed retrospectively using the HIRA database from 2013 to 2018, and the prevalence and risk factors for blood transfusion and postoperative infection associated with blood transfusion in lumbar spinal fusion were investigated. The present study showed an overall blood transfusion rate of 52.63% in lumbar spinal fusion. The identified independent risk factors associated with blood transfusion were older age, female sex, the posterior approach, osteotomy, and the presence of comorbidities.

The overall prevalence of blood transfusion in lumbar spinal fusion was 52.63%. Fortunately, this study showed a downward trend in the annual blood transfusion rates from 56.38% in 2013 to 47.51% in 2018. In previous studies, diverse results regarding the rate of blood transfusion in lumbar spinal fusion were observed, with overall blood transfusion rates reported from 36% to 75% [24,25].

Spine surgeons have used various strategies to minimize blood loss to reduce the complications associated with blood loss and blood transfusion [26]. Effectively predicting which patients are at an increased risk of greater intraoperative blood loss and are more likely to require perioperative blood transfusion provides surgeons with the knowledge for more optimized preoperative surgical planning to enhance the utilization of healthcare resources [27]. Patient blood management (PBM), which is defined as managing patients at risk of blood transfusion to reduce the need for blood transfusion and improve clinical outcome, can reduce blood transfusions. PBM is based on the correction of preoperative anemia, reducing intraoperative blood loss, and the optimization of anemia tolerance [15]. Implementing PBM results in better surgical outcomes and significant financial savings [28]. Minimal invasive surgery was also associated with decreased blood loss and reduced rates of blood transfusion [29,30].

The present study results also showed that several patient (age, sex, insurance type, presence of comorbidities), surgery (approach, osteotomy), and hospital (size, region) characteristics were significant risk factors for blood transfusion in lumbar spinal fusion. These results are consistent with the risk factors identified in the literature to be associated with blood transfusion. Therefore, we propose that these risk factors can be used by surgeons to inform patients who might need a blood transfusion as such and identify them as higher risk. With a better understanding of the potential risk factors for blood transfusion, efforts should focus on reducing the effects of these factors on postoperative outcomes and the need for blood transfusion in the future.

Older patients are more likely to receive blood transfusion because higher hemoglobin levels are necessary to maintain oxygen delivery to tissues [31]. A higher blood transfusion rate in females has been found in lumbar spinal fusion. A greater total comorbidity burden is an important risk factor for blood transfusion. The need for blood transfusion has been associated with the presence of comorbidities. Torres-Claramunt et al. showed that patients with an ASA score of 3 had an almost 18-fold increased risk of blood transfusion compared to those with a lower score [20].

The association between surgical approach and blood transfusion requirement was investigated in the present study. Patients who underwent the posterior approach were more likely to receive blood transfusion compared with subjects who underwent the anterior or anterior and posterior approach. The posterior approach requires large surgical exposure of the hypervascular paraspinal muscles, which can lead to massive blood loss [32]. Yoshihara et al. showed that the posterior approach was associated with a higher risk of blood transfusion, which is consistent with the results presented in the present study [4]. Butler et al. also reported the posterior approach to be associated with a greater risk of blood transfusion [33]. Our results are consistent with previous studies that have reported an association between three-column osteotomy and perioperative blood transfusion requirements, because osteotomy has the potential for extensive blood loss [34,35].

In addition to patient and surgical characteristics, specific hospital characteristics were associated with blood transfusion. Patients who underwent surgery in smaller hospitals were more likely to receive blood transfusion than those in larger hospitals. These results might reflect differences between hospitals in the implementation of blood-conserving strategies [19,36]. Surgeons with more experience can better manage preoperative hemoglobin and intraoperative blood loss in lumbar spinal fusion, reducing the need for blood transfusion. In particular, a significant difference was observed in the prevalence of blood transfusion based on region, with patients treated in a metropolitan area more than twice as likely to receive blood transfusion than subjects in a rural area.

The study results showed a significant increase in infection rates after lumbar spinal fusion in patients who received blood transfusions during the perioperative period. This observation can be explained by the immunosuppressive effect of blood transfusion [37,38]. Janssen et al. reviewed 3721 lumbar spine surgeries and found an increased OR of 2.2 for postoperative infections when comparing blood transfusion and no-transfusion groups [37]. Triulzi et al. reported that, among patients undergoing spinal fusion surgery, those who received transfusion were more likely to experience postoperative infection and a prolonged length of hospital stay compared with patients who did not [39]. Schwarzkopf et al. studied blood transfusions in lumbar surgery and found the infection group to have received more blood transfusions [40].

Because the present study was a retrospective analysis using large administrative databases, it was limited by several inherent factors. First, this study was based on claim data with limited accuracy of diagnostic and surgical codes. In this setting, significant error or inaccurate coding can occur. Because coding practices evolve over time, accuracy improves, allowing for a better understanding of blood transfusion rates. However, the correctness of this coding system has been validated in several previous studies. A second limitation is that this database does not contain detailed information such as perioperative hemoglobin values, pain, quality of life, function, neurological status, or surgical data such as operation time, amount of blood loss, level of fusion, or other immediate complications including mortality. Furthermore, the number of fusion levels, which is an important risk factor for blood transfusion, could not be determined. In addition, the database does not include information on preoperative patient optimization, pharmacologic interventions to reduce intraoperative blood loss, or the use of drugs that affect coagulation or platelet function.

Despite these limitations, the present study included large and diverse samples and provided reasonable patient, surgical, and hospital factors influencing the risk of blood transfusion, as well as the overall prevalence and trend of blood transfusion in patients undergoing lumbar spinal fusion. To the best of our knowledge, this is the first nationwide population-based analysis in which blood transfusion after lumbar spinal fusion was investigated.

Despite the successful results of lumbar spinal fusion, blood loss and the need for blood transfusion continue to burden patients and healthcare providers with poor outcomes and high costs. Fortunately, in this study, the annual prevalence of blood transfusion showed a downward trend from 56.38% in 2013 to 47.51% in 2018. Most comorbidities investigated were associated with an increased risk of blood transfusion, and patients who received blood transfusion had an increased risk of postoperative infections. Because the risks of blood transfusion have been emphasized in the literature, the decline in blood transfusion rate is very positive. As the frequency of lumbar spinal fusion is expected to increase, spine surgeons must be aware of how to effectively prevent, diagnose, and manage perioperative blood loss to minimize blood transfusions.

## 5. Conclusions

This study used a large database to identify the prevalence, risk factors, and rates of postoperative infection associated with blood transfusion in lumbar spinal fusion. These factors must be taken into consideration for patients’ blood management prior to surgery. Spine surgeons should consider these risk factors in patients at high risk of blood transfusion.

## Figures and Tables

**Table 1:** Incidence of blood transfusion among lumbar spinal fusion patients in Korea from 2013 to 2018.

	2013	2014	2015	2016	2017	2018	Total
Total	31,339	30,816	30,778	32,562	31,385	31,701	188,581
Transfusion (%)	17,669 (56.38%)	16,881 (54.78%)	16,701 (54.26%)	17,213 (52.86%)	15,721 (50.09%)	15,062 (47.51%)	99,247
Surgical approach							
Anterior	293	326	287	324	281	275	1786
Posterior	16,333	15,468	15,294	15,615	14,210	13,547	90,467
Anterior and posterior	1043	1087	1120	1274	1230	1240	6994

**Table 2:** Baseline characteristics and comorbidities.

	All Patients	Transfusion		*p*-Value *	Surgical Approach			*p*-Value *
		Transfusion	No Transfusion		Anterior	Posterior	Anterior and Posterior	
Total	188,581	99,247 (52.63)	89,334 (47.37)		4677 (2.48)	163,678 (86.79)	20,226 (10.73)	
Age, n (%)				<0.0001				<0.0001
<40	6208 (3.29)	2341 (2.36)	3867 (4.33)		493 (10.54)	5306 (3.24)	409 (2.02)	
40–49	11,938 (6.33)	4590 (4.62)	7348 (8.23)		685 (14.65)	10,011 (6.12)	1242 (6.14)	
50–59	41,049 (21.77)	17,557 (17.69)	23,492 (26.3)		1067 (22.81)	35,203 (21.51)	4779 (23.63)	
60–69	64,993 (34.46)	33,802 (34.06)	31,191 (34.92)		1232 (26.34)	56,360 (34.43)	7401 (36.59)	
≥70	64,393 (34.15)	40,957 (41.27)	23,436 (26.23)		1200 (25.66)	56,798 (34.70)	6395 (31.62)	
Gender, n (%)				<0.0001				<0.0001
Male	74,222 (39.36)	32,788 (33.04)	41,434 (46.38)		1948 (41.65)	65,753 (40.17)	6521 (32.24)	
Female	114,359 (60.64)	66,459 (66.96)	47,900 (53.62)		2729 (58.35)	97,925 (59.83)	13,705 (67.76)	
Insurance, n (%)				<0.0001				<0.0001
Health insurance	174,142 (92.34)	89,932 (90.61)	84,210 (94.26)		4423 (94.57)	150,535 (91.97)	19,184 (94.85)	
Medical Aid	14,439 (7.66)	9315 (9.39)	5124 (5.74)		254 (5.43)	13,143 (8.03)	1042 (5.15)	
Hospital size, n (%)				<0.0001				<0.0001
Tertiary referral hospital	48,249 (25.59)	25,302 (25.49)	22,947 (25.69)		1075 (22.98)	44,076 (26.93)	3098 (15.32)	
General hospital	50,935 (27.01)	28,595 (28.81)	22,340 (25.01)		651 (13.92)	45,862 (28.02)	4422 (21.86)	
Hospital	88,171 (46.75)	44,358 (44.69)	43,813 (49.04)		2941 (62.88)	72,533 (44.31)	12,697 (62.78)	
Private clinic	1226 (0.65)	992 (1.00)	234 (0.26)		10 (0.21)	1207 (0.74)	9 (0.04)	
Hospital region, n (%)				<0.0001				<0.0001
Metropolitan	119,419 (63.33)	60,360 (60.82)	59,059 (66.11)		2558 (54.69)	99,889 (61.03)	16,972 (83.91)	
Urban	68,820 (36.49)	38,617 (38.91)	30,203 (33.81)		2118 (45.29)	63,459 (38.77)	3243 (16.03)	
Rural	342 (0.18)	270 (0.27)	72 (0.08)		1 (0.02)	330 (0.20)	11 (0.05)	
Osteotomy, n (%)	432 (0.23)	419 (0.42)	13 (0.01)	<0.0001	164 (3.51)	204 (0.12)	64 (0.32)	<0.0001
Comorbidities, n (%)								
Myocardial infarction	4310 (2.29)	2580 (2.60)	1730 (1.94)	<0.0001	91 (1.95)	3818 (2.33)	401 (1.98)	0.0021
Congestive heart failure	19,006 (10.08)	11,557 (11.64)	7449 (8.34)	<0.0001	399 (8.53)	16,638 (10.17)	1969 (9.73)	0.0003
Peripheral vascular disease	68,943 (36.56)	38,516 (38.81)	30,427 (34.06)	<0.0001	1354 (28.95)	59,864 (36.57)	7725 (38.19)	<0.0001
Cerebrovascular disease	36,678 (19.45)	21,391 (21.55)	15,287 (17.11)	<0.0001	746 (15.95)	32,105 (19.61)	3827 (18.92)	<0.0001
Dementia	7321 (3.88)	4466 (4.50)	2855 (3.20)	<0.0001	146 (3.12)	6395 (3.91)	780 (3.86)	0.0229
Chronic pulmonary disease	103,938 (55.12)	55,845 (56.27)	48,093 (53.84)	<0.0001	2425 (51.85)	89,854 (54.90)	11,659 (57.64)	<0.0001
Rheumatologic disease	32,304 (17.13)	18,379 (18.52)	13,925 (15.59)	<0.0001	719 (15.37)	27,850 (17.02)	3735 (18.47)	<0.0001
Peptic ulcer disease	104,009 (55.15)	55,844 (56.27)	48,165 (53.92)	<0.0001	2411 (51.55)	89,795 (54.86)	11,803 (58.36)	<0.0001
Mild liver disease	87,983 (46.66)	46,747 (47.10)	41,236 (46.16)	<0.0001	2104 (44.99)	76,065 (46.47)	9814 (48.52)	<0.0001
Diabetes	78,273 (41.51)	43,946 (44.28)	34,327 (38.43)	<0.0001	1635 (34.96)	68,448 (41.82)	8190 (40.49)	<0.0001
Diabetes complications	32,444 (17.20)	19,092 (19.24)	13,352 (14.95)	<0.0001	621 (13.28)	28,691 (17.53)	3132 (15.49)	<0.0001
Hemiplegia or paraplegia	4326 (2.29)	2604 (2.62)	1722 (1.93)	<0.0001	129 (2.76)	3804 (2.32)	393 (1.94)	0.0003
Renal disease	7281 (3.86)	4635 (4.67)	2646 (2.96)	<0.0001	146 (3.12)	6509 (3.98)	626 (3.10)	<0.0001
Any malignancy including leukemia and lymphoma	14,540 (7.71)	8149 (8.21)	6391 (7.15)	<0.0001	291 (6.22)	12,851 (7.85)	1398 (6.91)	<0.0001
Severe liver disease	1529 (0.81)	945 (0.95)	584 (0.65)	<0.0001	38 (0.81)	1341 (0.82)	150 (0.74)	0.509
Metastatic solid tumor	1683 (0.89)	1154 (1.16)	529 (0.59)	<0.0001	46 (0.98)	1495 (0.91)	142 (0.70)	0.0085

* *p*-value by Chi-square test.

**Table 3:** Odds ratios (ORs) of blood transfusion based on confounding factors.

	OR	CI	*p*-Value
Surgical approach			
Anterior	0.511	(0.48, 0.545)	<0.0001
Posterior	ref		
Anterior and posterior	0.426	(0.412, 0.44)	<0.0001
Age			
<40	ref		
40–49	0.948	(0.888, 1.012)	0.1067
50–59	1.082	(1.022, 1.145)	0.0069
60–69	1.541	(1.457, 1.63)	<0.0001
≥70	2.391	(2.258, 2.531)	<0.0001
Gender			
Male	ref		
Female	1.772	(1.737, 1.807)	<0.0001
Insurance			
Health insurance	ref		
Medical Aid	1.534	(1.478, 1.592)	<0.0001
Hospital size			
Tertiary referral hospital	ref		
General hospital	1.115	(1.086, 1.146)	<0.0001
Hospital	1.03	(1.005, 1.055)	0.0166
Private clinic	4.148	(3.583, 4.801)	<0.0001
Hospital region			
Metropolitan	ref		
Urban	1.211	(1.186, 1.236)	<0.0001
Rural	3.506	(2.682, 4.582)	<0.0001
Osteotomy	37.019	(21.227, 64.558)	<0.0001
Comorbidities			
Myocardial infarction	1.149	(1.076, 1.226)	<0.0001
Congestive heart failure	1.119	(1.082, 1.157)	<0.0001
Peripheral vascular disease	1.001	(0.98, 1.023)	0.8932
Cerebrovascular disease	1.032	(1.005, 1.058)	0.0173
Dementia	1.016	(0.966, 1.069)	0.538
Chronic pulmonary disease	0.93	(0.911, 0.949)	<0.0001
Rheumatologic disease	1.08	(1.053, 1.109)	<0.0001
Peptic ulcer disease	0.951	(0.932, 0.971)	<0.0001
Mild liver disease	0.957	(0.937, 0.977)	<0.0001
Diabetes	1.032	(1.01, 1.056)	0.0048
Diabetes complications	1.101	(1.071, 1.133)	<0.0001
Hemiplegia or paraplegia	1.308	(1.226, 1.396)	<0.0001
Renal disease	1.323	(1.257, 1.393)	<0.0001
Any malignancy including leukemia and lymphoma	1.009	(0.972, 1.048)	0.6226
Severe liver disease	1.387	(1.244, 1.546)	<0.0001
Metastatic solid tumor	2.166	(1.937, 2.421)	<0.0001

OR: Odds ratio; CI: confidence interval.

**Table 4:** Odds ratios (ORs) of infection based on confounding factors.

	OR	CI	*p*-Value
Transfusion			
Transfusion	1.876	(1.815, 1.939)	<0.0001
No transfusion	ref		
Surgical approach			
Anterior	1.28	(1.164, 1.408)	<0.0001
Posterior	ref		
Anterior and posterior	1.118	(1.062, 1.177)	<0.0001
Age			
<40	ref		
40–49	0.879	(0.795, 0.973)	0.0126
50–59	0.843	(0.772, 0.921)	0.0001
60–69	0.819	(0.751, 0.893)	<0.0001
≥70	0.876	(0.802, 0.956)	0.0029
Gender			
Male	ref		
Female	0.72	(0.698, 0.743)	<0.0001
Insurance			
Health insurance	ref		
Medical Aid	1.05	(0.994, 1.109)	0.0835
Hospital size			
Tertiary referral hospital	ref		
General hospital	1.185	(1.137, 1.236)	<0.0001
Hospital	0.875	(0.841, 0.91)	<0.0001
Private clinic	2.235	(1.943, 2.572)	<0.0001
Hospital region			
Metropolitan	ref		
Urban	0.98	(0.948, 1.012)	0.217
Rural	1.189	(0.871, 1.622)	0.2749
Osteotomy	1.256	(0.969, 1.629)	0.0846
Comorbidities			
Myocardial infarction	1.006	(0.912, 1.109)	0.9097
Congestive heart failure	1.062	(1.009, 1.117)	0.0207
Peripheral vascular disease	1.001	(0.968, 1.036)	0.9361
Cerebrovascular disease	0.977	(0.938, 1.017)	0.2583
Dementia	0.97	(0.896, 1.05)	0.4467
Chronic pulmonary disease	0.984	(0.953, 1.017)	0.3493
Rheumatologic disease	1.021	(0.98, 1.064)	0.3234
Peptic ulcer disease	0.987	(0.955, 1.02)	0.4245
Mild liver disease	1.099	(1.063, 1.136)	<0.0001
Diabetes	1.075	(1.037, 1.114)	<0.0001
Diabetes complications	1.077	(1.031, 1.124)	0.0008
Hemiplegia or paraplegia	1.247	(1.139, 1.364)	<0.0001
Renal disease	1.002	(0.929, 1.081)	0.9501
Any malignancy including leukemia and lymphoma	0.929	(0.875, 0.986)	0.0157
Severe liver disease	0.907	(0.768, 1.07)	0.2462
Metastatic solid tumor	0.883	(0.748, 1.043)	0.1433

OR: Odds ratio; CI: confidence interval.

## Data Availability

The original contributions presented in the study are included in the article, further inquiries can be directed to the corresponding author/s.
